# Another Experiment in Articulation

**Published:** 1918-12-14

**Authors:** 


### THE COLLEGE PROGRAMME.

### ANOTHER EXPERIMENT IN ARTICULATION.

The College of Nursing has now been established for a very considerable time, during which period it has, with the help of the nursing press, won the confidence of ten thousand members. The College has lived through a storm of hostile criticism, and is, to-day, in a stronger position than at any previous time. Born of the war, both the College and its members have helped the armies of the Allies to victory—victory more complete and sudden than the most optimistic dared to hope. The question now arises—What is the College programme? I should much like to write an article setting this forth in detail; but I am unable to do so because I do not know. I have been waiting, and waiting impatiently, for a trumpet note of policy, vigorous and immediate, for I am persuaded that if the nurse is to triumph with the nation there is no time to lose in contemplation. It is a case of to be or not to be, and the hour for decision is now striking.

### Two Outstanding Facts.

I am confronted with two outstanding facts. One is that ten thousand working nurses have paid their guineas in view of *something* which they have never expressed. The inarticulate nurse, sick unto death of faction and stagnation, speculated in faith, and joined up. So far she has derived no benefit which can be expressed in £ s. d. College Centres have been established, scholarships have been founded, and a nation's tribute has been set on foot. All this is excellent; but, I repeat, it is mainly so far insubstantial and intangible. The College of Nursing members are no better off because of their membership than ever they were, unless to have developed a corporate feeling is something worth putting on record. This is one fact. The other fact is that the elected representatives of the nurses who belong to the College have issued no specific manifesto, jointly or individually, of what they hope to achieve for their constituents.

### Crank, Outsider, or Idealist?

I am not satisfied. I may be a crank, or an "outsider," or an idealist. I do not either know or care to which category I may be relegated by friend or foe. I may be like the client of a certain solicitor of my acquaintance. The gentleman referred to was always extravagant, always unreasonable. He was bequeathed a small legacy, and, being impecunious, paid many visits to his attorney, who offered divers explanations of legal procedure and unavoidable delay. To these arguments he always listened, but always answered: "Yes, yes! I know: I understand. But when do I touch?" With complete faith in the potentiality of the College to accomplish the emancipation of the nurse I am not satisfied with recorded progress. There does not appear to me any outward and visible sign that the nurse is to "touch" very much in the immediate future. I do not like the silence.

### Leaders and their Antics.

It is my fate to be suspicious. I cannot help it, and, to be truthful, I do not try. I had been watching the leaders of nurses and their antics for many years before the College came into being, and it always appeared to me that nothing ensued but empty clatter. Cliques of avowed champions and leaders gathered together in parlours and talked. They called in lawyers and physicians and members of Parliament, and continued to talk. What did they *do*? And if they did anything and everything, the question still remains, has the nurse "touched"? As they say in Parliament, the answer is in the negative. She still works at a beggarly wage. Her hours of toil are out of all comparison with those of other workers, male or female. Her occupation is precarious. She has no financial outlook. And yet this woman is an indispensable worker for the richest Empire in the world. She has been an important factor in winning the war. She has helped to bring back to health tens of thousands of our sailors and soldiers. What I want to see is a strong committee set up to deal with its economic problem—it is already promised—and then the College Executive backing its recommendations.

### In Quest of the Inarticulate Nurse.

Now I am setting forth on a quest of my own. I propose to endeavour to discover what is in the mind of the inarticulate nurse, and to publish this for the information of all whom it may concern. There are ten thousand nurses who joined the College of Nursing, and I want to ask these what they expect the Executive of the College to accomplish for them. I am convinced that they had *something* in view when they joined, and so far as I am aware, they have never put their thoughts into words. I am not aware that they have ever been asked.

### An Apostle of Economics.

I am an apostle of economics, and at the same time a believer in the Halo. But I hold that both one and other are intimately associated. The nurse is out for a career, and she is also out for honour. She is often doing greater and better service for the nation and for the world than the committeemen who employ her. And, holding these views, I consider that she must be adequately paid. She must not be worked to death. College advocates, so far as I have been able to observe, and I have watched carefully, must not fight shy of the economic problem. They must not deal in generalities, and dwell exclusively on the importance of education. Let them come out for a living wage, security of employment, and generous pensions after years of service. Possibly the working nurse may agree: possibly not. I propose to try the experiment of finding out. And therefore I ask my readers to write to me in terms frank and free. Tell me, in brief, what you expect the College of Nursing to accomplish. Do not be led or guided by any sentiment of mine, true or false, loyal or disloyal. State your views without bias. Your confession will help the College by supplying a fund of information which it has no other means of ascertaining. Your letters will correspond with a lawyer's brief. The College, if it is anything, is a Nurse's Parliament.

### An Inviolate Sanctum.

Needless to observe, the Editor's sanctum is inviolate. He must be satisfied that his correspondents are *bona-fide* nurses, but with this proviso, no names, addresses, or hospitals will be mentioned without authorisation. I am concerned with one only consideration, which is that I am voicing the trained nurse. It is imperative that her voice shall be heard. And let it be understood that I am a believer in the College—its out-and-out supporter and champion, but keenly alive at the same time as to what may ensue if the British nurse should miss the tide.

IERNE.

